# The Influence of Astaxanthin on the Proliferation of Adipose-derived Mesenchymal Stem Cells in Gelatin-Methacryloyl (GelMA) Hydrogels

**DOI:** 10.3390/ma12152416

**Published:** 2019-07-29

**Authors:** Bo Young Choi, Elna Paul Chalisserry, Myoung Hwan Kim, Hyun Wook Kang, Il-Whan Choi, Seung Yun Nam

**Affiliations:** 1 Interdisciplinary Program of Biomedical Mechanical & Electrical Engineering, Pukyong National University, Busan 48513, Korea; 2 Center for Marine-Integrated Biomedical Technology (BK21 Plus), Pukyong National University, Busan 48513, Korea; 3 Department of Biomedical Engineering, Pukyong National University, Busan 48513, Korea; 4 Department of Microbiology, Inje University College of Medicine, Busan 48513, Korea

**Keywords:** Astaxanthin, Mesenchymal stem cells, Proliferation, Gelatin methacryloyl, Hydrogel, Tissue engineering

## Abstract

Recently, astaxanthin, a red lipophilic pigment belonging to the xanthophyllic family of carotenoids, has shown the feasibility of its uses in tissue engineering and regenerative medicine, due to its excellent antioxidant activities and its abilities to enhance the self-renewal potency of stem cells. In this study, we demonstrate the influence of astaxanthin on the proliferation of adipose-derived mesenchymal stem cells in tissue-engineered constructs. The tissue engineered scaffolds were fabricated using photopolymerizable gelatin methacryloyl (GelMA) with different concentrations of astaxanthin. The effects of astaxanthin on cellular proliferation in two-dimensional environments were assessed using alamar blue assay and reverse transcription polymerase chain reaction (RT-PCR). Then, rheological properties, chemical structures and the water absorption of the fabricated astaxanthin-incorporated GelMA hydrogels were characterized using NMR analysis, rheological analysis and a swelling ratio test. Finally, the influence in three-dimensional environments of astaxanthin-incorporated GelMA hydrogels on the proliferative potentials of adipose-derived stem cells was assessed using alamar blue assay and the confocal imaging with Live/dead staining. The experimental results of the study indicate that an addition of astaxanthin promises to induce stem cell potency via proliferation, and that it can be a useful tool for a three-dimensional culture system and various tissue engineering applications.

## 1. Introduction

Recently, bioprospecting marine-based biomaterials for tissue engineering applications have gained momentum due to a wide array of bioactivity, availability and economic viability [1]. Astaxanthin (3, 3’-dihydroxy-β, β-carotene-4, 4’-dione), a red lipophilic pigment belonging to the xanthophyllic family of carotenoids, can be derived from marine microorganisms and species such as the microalgae *Haematococcus pluvialis*. Its unique structure of the ketone, hydroxyl groups and the polyene chains can convert unstable electrons into a stable state and then terminate the free radical chain in the living organism to exhibit antioxidant activities. 

It therefore has beneficial properties, such as anti-inflammatory activity, by blocking the NF-κB dependent signaling pathway and immune enhancement by scavenging oxygen radicals [2,3]. It has also been shown that astaxanthin can be used for a broad spectrum of pharmacological and therapeutic applications, due to its outstanding anti-cancer and anti-apoptotic properties compared to other antioxidant carotenoids. Recent studies demonstrated that astaxanthin can change stem cell potency through the PI3K and MEK signaling pathways [4,5]. Particularly, it can enhance the differentiation of adipose-derived mesenchymal stem cells into oligodendrocytes, osteoblasts, chondrocytes and adipocytes [6,7,8]. These studies indicate the feasibility of the use of astaxanthin in various applications of tissue engineering and regenerative medicine.

Tissue-engineered three-dimensional scaffolds allow for a uniform interaction of bioactive metabolite with native cells providing a close resemblance of the in vivo microenvironment. Gelatin methacryloyl (GelMA) has been extensively used in tissue engineering applications requiring in vivo mimicry and three-dimensional cell culture. GelMA has RGD sites (Arg-Gly-Asp) for cell attachment, sequences of matrix metalloproteinase for biodegradability, excellent biocompatibility and tunable physical properties. As a photopolymer, it forms covalently cross-linked hydrogels when exposed to ultraviolet (UV) light in the presence of photoinitiators such as Lithium phenyl-2,4,6-trimethylbenzoylphosphinate (LAP) and 2-hydroxy-4’-(2-hydroxyethoxy)-2-methylpropiophenone (Irgacure 2959) [9,10,11,12,13,14,15,16]. Promising results have been observed, in particular, with stem cell proliferation, cellular migration and viability, as it mimics the natural extracellular matrix (ECM) by providing a suitable niche and microenvironment. Many studies in tissue engineering and regenerative medicine have shown that stem cells encapsulated in three-dimensional GelMA scaffolds can be used for the treatment of various diseases and have exhibited positive impacts [17,18,19,20]. Among the various stem cell types, adipose-derived mesenchymal stem cells (ADMSCs) display superior properties over other stem cells, namely: Easy isolation, minimal invasiveness, great safety, no immune rejection with autologous cells and a self-renewal capacity [21,22].

In this study, we hypothesize that astaxanthin increases the cell proliferation of ADMSCs in three-dimensional environments. The objective is to study the effects of astaxanthin on the proliferative potential of ADMSCs incorporated with GelMA. First, the astaxanthin was tested in two-dimensional environments to assess the cellular proliferation and stemness-related gene expression, with and without astaxanthin, using alamar blue assay and RT-PCR analysis. In addition, three-dimensional GelMA scaffolds with and without astaxanthin were fabricated and physiochemically characterized by NMR analysis, rheological analysis and a water absorption test. Finally, cellular proliferation and behavior in the astaxanthin-combined GelMA scaffolds were assessed using alamar blue assay and confocal imaging with Live/dead staining.

## 2. Results and Discussions

### 2.1. Cellular Proliferation Test in Two-Dimensional Environments

**Figure 1:**
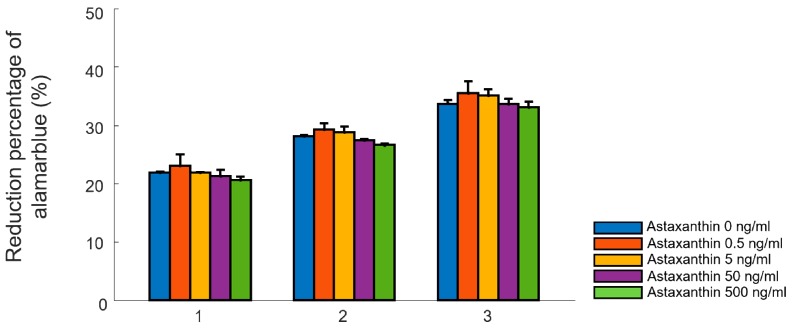
Proliferation test on adipose-derived mesenchymal stem cells in vitro. Cellular proliferation of adipose-derived mesenchymal stem cells treated with astaxanthin at various concentrations (0, 0.5, 5, 50, and 500 ng/ml) was assessed on day 1, 2, and 3 using alamar blue assay. Data are presented as the mean ± standard deviation (n = 3).

Figure 1 illustrates the effect of various concentrations of astaxanthin (0, 0.5, 5, 50, and 500 ng/ml) upon cellular proliferation. An overall increase in cell proliferation was seen across all groups in a time-dependent manner. In specific, the ADMSCs in the concentration of 0.5 ng/ml showed the highest proliferation (Figure 1). A decrease in cellular proliferation was observed in higher concentrations (500 ng/ml) with time. In addition, ADMSCs were treated with astaxanthin to support quantitative results at various concentrations as shown in Figure S1. Therefore, the astaxanthin concentration of 0.5 ng/ml was determined as the optimal concentration for further tests.

### 2.2. Reverse Transcription-Polymerase Chain Reaction (RT-PCR)

**Figure 2:**
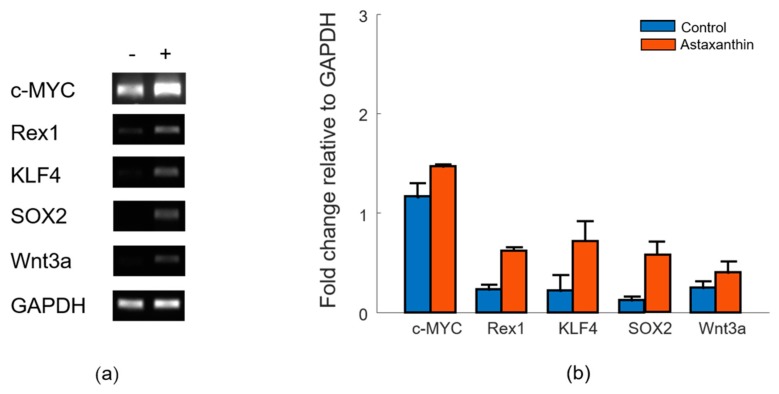
RT-PCR of adipose-derived mesenchymal stem cells cultured with and without astaxanthin in vitro. (**a**) Astaxanthin (0.5 ng/ml)-enhanced expression that related proliferation and cell renewal. (**b**) Quantified gene expression in bar graph normalized to GAPDH. Data are presented as the mean ± standard deviation (n = 3).

In various other studies, it has been demonstrated that multiple antioxidants such as N-acetyl-L-cysteine, L-ascorbic acid 2-phosphate, sulforaphane and epigallocatechin gallate have shown positive influences on cellular proliferation, as well as the stemness of stem cells [23,24,25]. Antioxidants can improve genomic stability and also promote proliferation by raising the number of cells in the S phage of the ADMSCs [26]. To evaluate the influence of astaxanthin, which has antioxidant properties on the proliferation of ADMSCs, reverse transcription-PCR (RT-PCR) for the expression of molecular markers, including proliferation-related transcription factors and stemness genes, was performed. As shown in Figure 2a,b, the treatment of astaxanthin in the concentration of 0.5 ng/ml for one day increases the upregulation of stemness genes (SOX2 and KLF4) and proliferation-related transcription factors (Rex1, c-MYC, and Wnt3a) [5]. In particular, the SOX2 gene was markedly over expressed, which is known for its role in the maintenance of pluripotency. c-MYC, known to transcriptionally amplify SOX2 target genes to regulate the self-renewal capacity of stem cells, was also upregulated [27]. Rex1 is critically important for maintaining proliferation in mesenchymal stem cells through the suppression of p38 MAPK signaling via the direct suppression of MKK3 [28]. ADMSCs treated with 0.5 ng/ml of astaxanthin indicated an overexpression of Rex1 genes because of the increased proliferation. Wnt3a was also upregulated, which indicated cellular proliferation was promoted through the wingless-related integration site (Wnt) signaling pathway [29]. Overexpression of KLF4, a direct target of the MAPK signaling pathway, improves the stemness of stem cells for self-renewal. Therefore, these results show that astaxanthin can enhance cellular proliferation and maintain cell stemness.

### 2.3. Characterization of Astaxanthin-Incorporated GelMA Scaffolds

#### 2.3.1. Fabrication of Astaxanthin-Incorporated GelMA Scaffolds

**Figure 3:**
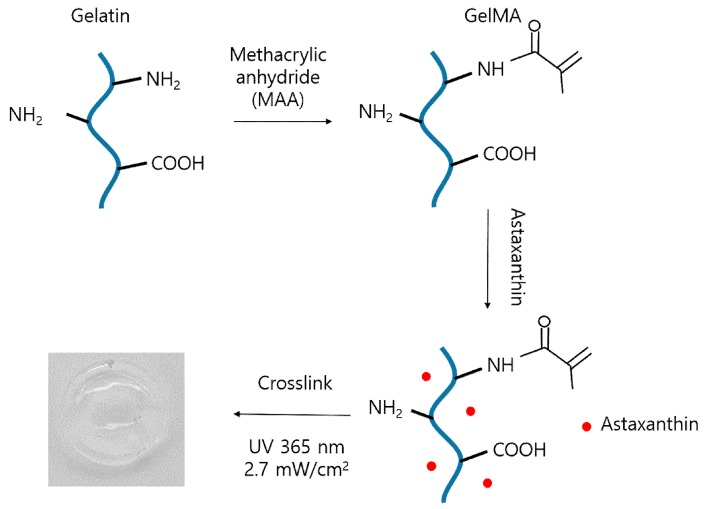
Schematic image of the fabrication of astaxanthin-incorporated gelatin methacryloyl (GelMA) scaffolds.

Gelatin was reacted with methacrylic anhydride to graft methacryloyl groups onto amine and hydroxyl groups as seen in Figure 3. Then, modified-gelatin solution was incorporated with astaxanthin. Finally, GelMA scaffolds combined with astaxanthin were fabricated by UV irradiation to crosslink polymeric networks using photoinitiator (Irgacure 2959).

#### 2.3.2. Degree of Methacrylation

The degree of substitution of free amino groups in samples can be modulated by the amount of methacrylic anhydride and the reaction times for photopolymerization. The proton nuclear magnetic resonance (^1^H NMR) confirms the peaks of the synthesized hydrogen atoms in methacrylate groups.

**Figure 4:**
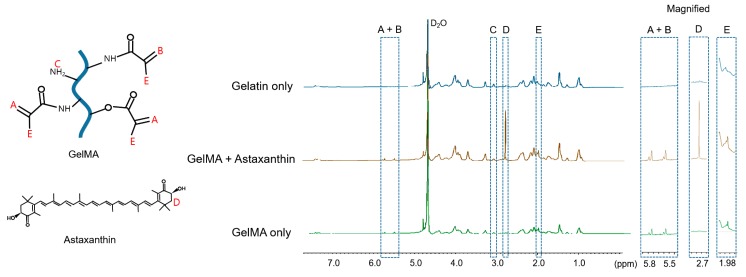
^1^H-NMR spectra of gelatin only, GelMA only, and GelMA incorporated with astaxanthin in D_2_O. Some specific protons were shown as follows: Box A+B-acrylic protons (2H) of methacryloyl functions. Box C-methylene protons (2H) of non-modified lysine groups. Box D-astaxanthin peak. Box E-methyl protons (3H) groups of methacryloyl groups.

For the quantification of the degree of substitution, the spectra were normalized to the phenylalanine signal (7.0–7.5 ppm), which represents the concentrations of gelatin. New peaks (5.5 and 5.8 ppm) appeared in the spectrum of GelMA comparing to that of gelatin, which indicates the acrylic protons (2H) of methacrylic methacryloyl functions (box A + B in Figure 4). The peaks of GelMA with and without astaxanthin shown in box C were corresponded to the methylene protons (2H) of unreacted lysine groups (3.1 ppm) and were slightly decreased compared to that of gelatin. Also, in box D, the peak was observed only in GelMA with astaxanthin (2.7–2.8 ppm), indicating the presence of astaxanthin [30,31]. The peaks in box E (1.98 ppm) for the methyl protons (3H) groups of methacrylamide grafts show methacrylation in GelMA. The phenylalanine peaks (7.0–7.5 ppm) were set as the internal reference to normalize the amine signals (3.1 ppm) of methacrylated lysine. The degree of substitution with the methacrylate group used in this study was 56.65% yield by the ^1^H NMR spectrum [11,32,33]. Therefore, the methacrylate groups have been successfully grafted to the gelatin as shown in box A + B, C, and E well incorporated with astaxanthin in GelMA as presented in box D.

#### 2.3.3. Rheological Characterization 

**Figure 5:**
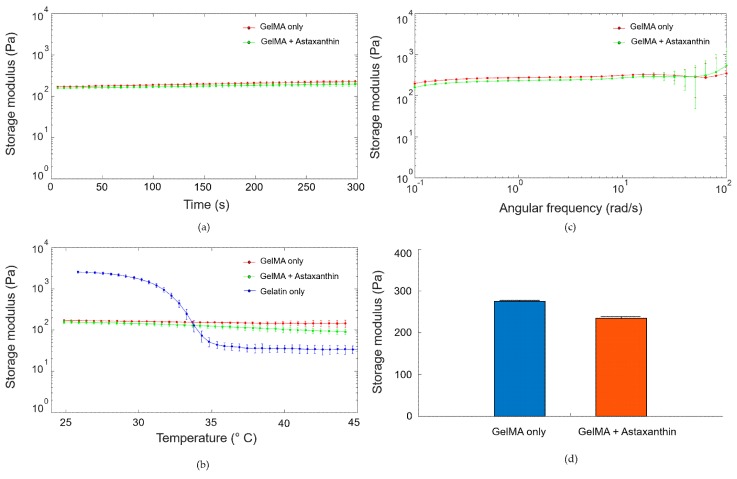
Rheological properties of GelMA with and without astaxanthin (**a**) time sweep test, (**b**) temperature ramp test, (**c**) frequency sweep test, and (**d**) the values of storage moduli (G’) at the frequency of 1 rad/s. Data are presented as the mean ± standard deviation (n = 3).

Before the assessment of storage moduli, strain amplitude sweeps (0.01–100%) were first performed to choose the linear viscoelastic region. A time-sweep test was performed to confirm that the object is maintaining its time-stable form for five minutes. All of the samples maintained its storage moduli in the time-dependent experiment as shown in Figure 5a. In the temperature-dependent rheological test in Figure 5b, the storage moduli of GelMA samples with and without astaxanthin decrease very slightly as the temperature increases (red and green). However, as thermo-sensitive polymer, the storage modulus of gelatin (blue) was dramatically reduced over 30 °C [16]. These results elucidated that GelMA-based hydrogels withstood gradient temperature conditions better than gelatin hydrogels because of a more robust networking of crosslinking. GelMA showed slightly decreased storage moduli (G’) in a frequency sweep test in Figure 5c,d (GelMA only: 275 ± 1 Pa and GelMA with astaxanthin: 230 ± 1.9 Pa at 1 rad/s) frequency range possibly due to the scavenging property of astaxanthin thereby reducing free radicals generation.

#### 2.3.4. Swelling Behaviors

**Figure 6:**
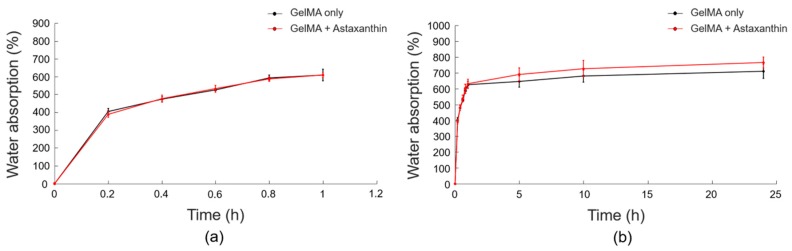
The swelling behaviors of GelMA with astaxanthin (red) and without astaxanthin (black) (**a**) from 0 to 1 hour and (**b**) from 0 to 24 hours. Data are presented as the mean ± standard deviation (n = 4).

In hydrogel, the swelling ability is an essential aspect of tissue engineering, since it affects various parameters including surface properties, mobility and solute diffusion [13]. The swelling behaviors of GelMA with astaxanthin (red) and without astaxanthin (black) are shown in Figure 6. GelMA with 0.5 ng/ml of astaxanthin exhibited a more increased swelling ratio than did the control. Specifically, the swelling ratio of all samples increased during the initial 1 h, and almost reached an equilibrium after 5 h of immersion. Water absorption in hydrogels was reduced significantly after 1 h. This can be attributed to the antioxidant property of astaxanthin, wherein the ratio of radical polymerization of the macromers was reduced by quenching the radical formed, as demonstrated by Chiellini et al. [34]. The experimental results may indicate astaxanthin containing ketone and hydroxyl groups possibly scavenges the free radicals in GelMA, which can make an effect of a slight increase of water absorption.

### 2.4. Cellular Proliferation in Astaxanthin-Incorporated GelMA Scaffolds

**Figure 7:**
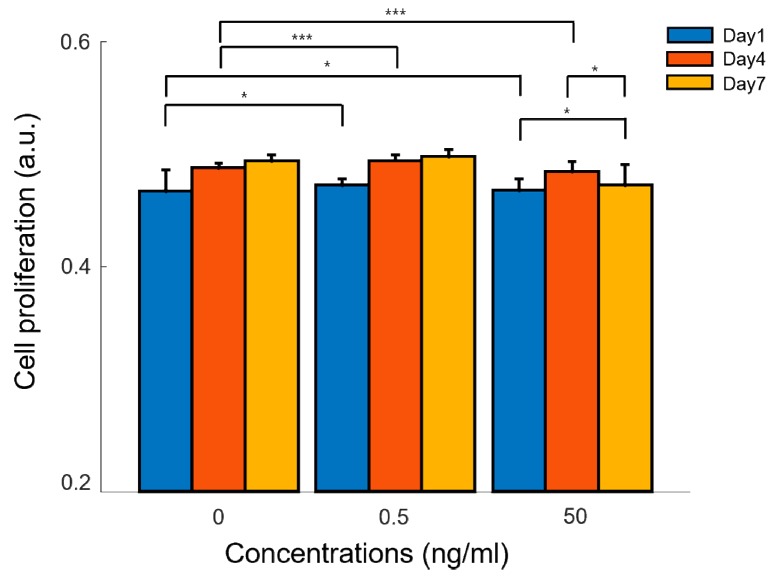
Cellular proliferation in three-dimensional GelMA-based hydrogels with different concentrations of astaxanthin (0, 0.5, and 50 ng/ml) on day 1, 4, and 7. Data are presented as the mean ± standard deviation (n = 4). (***p < 0.001, *p < 0.05).

The alamar blue assay investigated the cellular proliferation of adipose-derived mesenchymal stem cells encapsulated in GelMA hydrogels with astaxanthin (0.5 ng/ml and 50 ng/ml) and without astaxanthin (control) on day 1, 4, and 7 (Figure 7). The control graph shows an increase in cellular proliferation over time, which implies a non-toxicity and a cell attachment of GelMA-based hydrogels. Compared to our control, astaxanthin incorporated into GelMA significantly enhanced the cellular proliferation of ADMSCs on day 4 for both concentrations (0.5 ng/ml and 50 ng/ml). However, a slight dip was noticed on day 7 in 50 ng/ml of astaxanthin, which could be overdosed to promote cellular proliferation. ADMSCs cultured in three-dimensional GelMA-based hydrogel also reflected that 0.5 ng/ml of astaxanthin was the optimal concentration at different time points as observed in the two-dimensional cell culture experiment.

### 2.5. Confocal Imaging of ADMSCs in GelMA Hydrogels

**Figure 8:**
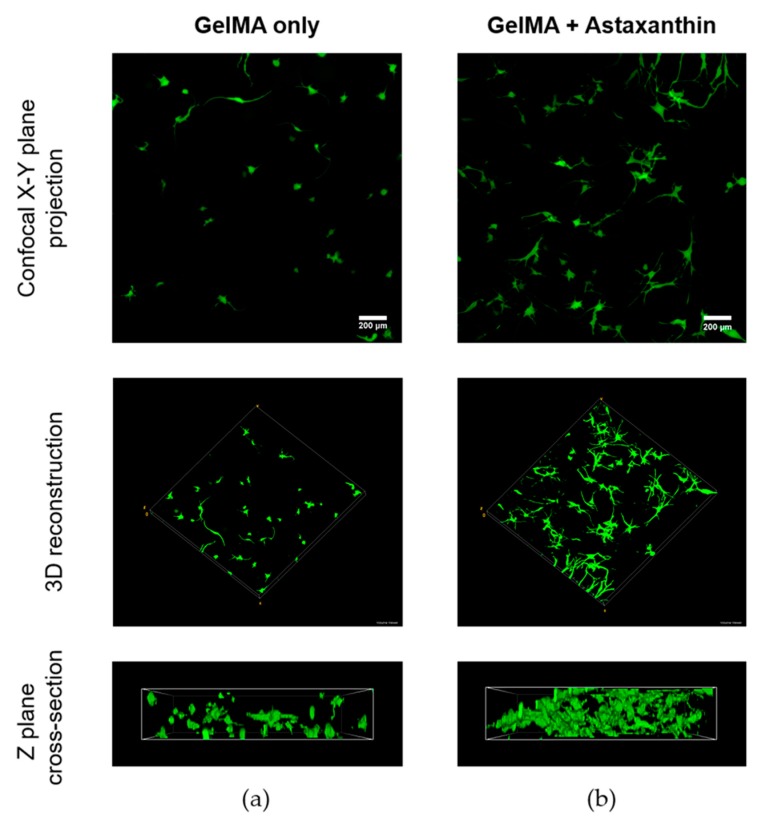
Confocal images of adipose-derived mesenchymal stem cells (ADMSCs) in GelMA (**a**) with and (**b**) without astaxanthin (0.5 ng/ml) using Live/dead staining after 8 days. (scale bar = 200 μm).

Figure 8 shows confocal images of the ADMSCs in 5% GelMA hydrogels using Live/dead staining to demonstrate the difference of cell viability with (Figure 8b) and without the interaction with astaxanthin (Figure 8a) after 8 days. The dimensions of all samples are 10 mm in diameter and 2 mm in height. The width, depth and height of the scanning area of the confocal microscope are 1272.79 μm, 276 μm, and 1272.79 μm, respectively. The first row of the images of Figure 8 clearly shows that ADMSCs thrived in both the hydrogels, and that the photo-crosslinking treatment of the hydrogel did not produce any significant deleterious effects on cell viability. In Figure 8, there is an obvious increase in the number of cells with an even spread in the GelMA hydrogels with astaxanthin compared to the control without astaxanthin. Evidently, ADMSCs treated with astaxanthin show more robust cell morphology with elongation. Moreover, more filopodia were observed in Figure 8b, indicating an early interconnected network of cells within the hydrogel. Also in other studies, it has been demonstrated that major carotenoids including astaxanthin, carotene, lutein, zeaxanthin and lycopene stimulate gap junctional intercellular communication, changing the phosphorylation pattern of connexin [35,36]. This could be attributed to the presence of astaxanthin in a three-dimensional hydrogel which induces improved cell-cell communication, thus exhibiting a desirable trait in three-dimensional scaffolds that mimics in vivo environments [18,19].

## 3. Materials and Methods 

### 3.1. Cell Culture with Astaxanthin Reagent

Astaxanthin (3, 3’-dihydroxy-β, β-carotene-4, 4’-dione) derived from the algae *Haematococcus Pluvialis* (Sigma-Aldrich, MO, IL, USA) was solubilized in dimethyl sulfoxide (DMSO) [37]. A stock solution of astaxanthin at 10 mg/ml were prepared and filtered with 0.22 μm nylon membrane filter (Jet biofil, Guangzhou, China). Further, astaxanthin at various concentrations (i.e., 0, 0.5, 5, 50, and 500 ng/ml) were studied in vitro for cell proliferation. Rabbit adipose-derived mesenchymal stem cells (Cyagen, Guangdong, China) were cultured in a basal culture medium of Dulbecco’s Modified Eagle’s medium (DMEM), supplemented with 4.5g/L glucose, L-glutamine, sodium pyruvate, 10% fetal bovine serum (FBS), 1% penicillin-streptomycin, and 1% non-essential amino acids. Cells were incubated in a 5% CO_2_ incubator at 37 °C and 95% humidity. Passages of 5 and 6 were used in all experiments.

### 3.2. GelMA Synthesis 

Briefly, a 10% (w/v) gelatin solution of type A (porcine, Bloom strength ~300, Sigma-Aldrich, USA) was dissolved under constant stirring in Dulbecco’s phosphate buffered saline (PBS) at 50 °C. Further, methacrylic anhydride (Sigma-Aldrich, MO, IL, USA) was added to gelatin solution at 50 °C. Post rigorous stirring for 1 hour, the reaction was stopped with PBS (5×). The mixture was then dialyzed through a 14 kDa molecular-weight-cutoff (MWCO) membrane (Sigma-Aldrich, MO, IL, USA) at 40 °C for 5 days against ultrapure water. After discarding the by-products, the samples were lyophilized for 3–4 days and stored at −20 °C until further use [9]. In all experiments, 5% of gelatin methacryloyl (GelMA) were used.

### 3.3. Fabrication of GelMA Scaffolds with Mesenchymal Stem Cells

The cultured adipose-derived mesenchymal stem cells (ADMSCs) were trypsinized, counted, and spun down. The resulting pellets were introduced to 5% of GelMA pre-solution and 0.5% (w/v) photoinitiator of Irgacure 2959 (Sigma-Aldrich, MO, IL, USA); sterilized with 0.22 μm polyethersulfone (PES) membrane syringe filter (Jet biofil, Guangzhou, China). Finally, the material was crosslinked using a 365 nm ultraviolet (UV) lamp (Thorlabs, USA) with a light intensity of 2.7 mW/cm^2^ for 60 sec and washed 2 times before culturing the hydrogels in DMEM at 37 °C and 5%CO_2_. 

### 3.4. Alamar Blue Assay

1 × 10^4^ cells/ml ADMSCs were treated with the above-mentioned concentrations of astaxanthin solution in DMEM and cultured at 37 °C and 5% CO_2_. Cell proliferation was assessed at 1, 2, and 3 days using the alamar blue reagents (Invitrogen, Carlsbad, CA, USA) in two-dimensional culture. A 10% alamar blue reagent was directly added to the ADMSCs encapsulated hydrogels with astaxanthin and without astaxanthin for 4 hours after day 1, 4, and 7. Absorbances were measured using a UV-Vis spectrophotometer (BioTek, Winooski, VT, USA) at 570 nm and 600 nm. All proliferation assays were performed in at least three separate experiments for each day. The percent reduction of alamar blue was calculated using the following equation:

(1) $$\mathrm{Percentage}\;\mathrm{reduction}\;\left(\%\right)=\left(\frac{\left(O_{2}\times A_{1}\right)-\left(O_{1}\times A_{2}\right)}{\left(R_{1}\times N_{2}\right)-\left(R_{2}\times N_{1}\right)}\right)\times 100$$

where, Q_1_ = the molar extinction coefficient (E) of oxidized alamar blue at 570 nm; Q_2_ = E of the oxidized alamar blue at 600 nm. R_1_= E of the reduced alamar blue at 570 nm; R_2_ = E of reduced alamar blue at 600 nm; A_1_ = absorbance of test wells at 570 nm; A_2_ = absorbance of test wells at 600 nm; N_1_ = absorbance of negative control (without cells) at 570 nm; N_2_ = absorbance of negative control (without cells) at 600 nm.

### 3.5. RNA Extraction and RT-PCR

Post 24 hours exposure of ADMSCs with 0 and 0.5 ng/ml of astaxanthin, the total RNA was isolated using TRI regent (Sigma-Aldrich, MO, IL, USA). The total RNA concentration was measured using UV/Vis-spectrophotometry (BioTek, Winooski, VT, USA) at 260 nm. cDNA was synthesized from the RNA using the reverse transcription method PrimeScript 1^st^ strand cDNA Synthesis Kit (Takara, Shiga, Japan). Template DNA was then used in gene-specific PCR, wherein synthesized cDNA using oligo-dT primer was amplified by 40 cycles (initial denaturation, denaturation, annealing, and extension: 98 °C, 1 min, 98 °C, 10 sec; 55–60 °C, 30 sec; 72 °C, 1 min). The expression of stemness-related genes (SOX2 and KLF4 (Bioneer, Alameda, California, USA)) and proliferation-related genes (Rex1, c-MYC, and Wnt3a, (Bioneer, Alameda, California, USA)) were studied, wherein Glyceraldehyde 3-phosphate dehydrogenase (GAPDH) was utilized as a housekeeping gene. Details of the primers are listed in Table 1. Further, aliquots of PCR product were electrophoresed on 1.5% agarose gels, PCR fragments were stained by loading STAR dye (Dynebio, Gyeonggi-do, South Korea) and detected by the gel documentation system (Daihan Scientific, Seoul, South Korea). All gene expression experiments were performed in triplicates.

### 3.6. Proton Nuclear Magnetic Resonance

The lyophilized GelMA hydrogel with and without astaxanthin were dissolved using deuterium oxide (D_2_O) solution at 40 °C. The methacrylation degree of the free amine group in the GelMA samples were studied using the Fourier transform nuclear magnetic resonance spectrometer JNM ECP-600 MHz (JEOL, Tokyo, Japan). The data were processed using JEOL delta V5.3 software (JEOL, Tokyo, Japan) and the degree of methacrylation was calculated as follows [11]:

(2) $$\mathrm{Methacrylation}\;\mathrm{degree}=\left(1-\frac{\mathrm{Lysine}\;\mathrm{integration}\;\mathrm{signal}\;\mathrm{of}\;\mathrm{GelMA}\;}{\mathrm{Lysine}\;\mathrm{integration}\;\mathrm{signal}\;\mathrm{of}\;\mathrm{gelatin}}\right)\times 100\;$$

### 3.7. Rheological Assessments 

A dynamic rheological test of the hydrogels was performed (Discovery HR-2, TA instrument, USA) to analyze the mechanical property of GelMA and GelMA/astaxanthin hydrogels using a rheometer equipped with an 8 mm parallel plate. The samples were irradiated with UV for 60 s and loaded into a 1mm gap. Strain amplitude sweeps (0.01–100%) were first performed to determine the linear viscoelasticity region. A time sweep test was set at an angular frequency of 10 rad/s for 300 s to study the time-dependent stability of the material. The frequency sweep test was performed at an angular frequency of 0.01–100 rad/s at a strain in the linear viscoelastic ranging to protect against destroying the structure of the samples. All tests were performed at 25 °C with a fixed strain of 1%, except the temperature ramp test, which ranged between 45 to 25 °C and demonstrated in triplicate [15,38].

### 3.8. Swelling Ratio Test 

For mechanical characteristics and the diffusion process, the hydrogels were lyophilized under dry conditions and were weighed (W_d_). The dried hydrogels were then immersed in PBS, incubated at 37 °C and weighed (W_s_). The swelling ratio was calculated under this formula:

(3) $$\mathrm{Swelling}\;\mathrm{ratio}\;\left(\%\right)=\frac{\left(\mathrm{Ws}\;-\;\mathrm{Wd}\right)\;}{\mathrm{Wd}}\times 100$$

where, W_d_ is the dry weight of hydrogel, W_s_ is the weight of swollen hydrogel.

### 3.9. Confocal Imaging Using Live/Dead Staining

Qualitative analysis and morphological variations of cell proliferation were carried by staining the encapsulated hydrogel with and without astaxanthin. Briefly the cells were incubated in fluorescein diacetate (FDA, Sigma-Aldrich, USA) and propidium iodide (PI, Sigma-Aldrich, USA) dyes for 6 min at room temperature and were then washed with PBS thrice. All three-dimensional images were scanned by a confocal laser scanning microscope system A1+ (Nikon, Tokyo, Japan) at 100× magnification. The Live/dead images were compared and stacked to z-projection and volume using the ImageJ software (V1.8, NIH, Bethesda, MD, USA) and an NIS-Elements viewer V4.50 software (Nikon, Japan).

### 3.10. Statistical Analysis

Data were analyzed as mean ± standard deviation. Difference between experimental groups were evaluated with a one-way analysis of variance (ANOVA), and the level of significance was set at *p* < 0.05, 0.01, and 0.001 (labeling *, **, and *** respectively). All analyses were performed by IBM SPSS Statistics Version 12.0 (SPSS, Chicago, IL, USA).

## 4. Conclusions

In this study, we demonstrated that astaxanthin had a positive impact upon the cell proliferation of adipose-derived mesenchymal stem cells in a time dependent manner. Thereafter, the upregulated proliferation-related transcription factors coupled with an overexpression of stemness genes confirmed the role of astaxanthin in improving the stem cell potency. 

Furthermore, physico-chemical characterization and biological analysis of hydrogels shows the feasibility of encapsulated GelMA hydrogel with astaxanthin to enhance cell-to-cell networking. Thus, the addition of astaxanthin promises to induce stem cell potency via proliferation, and it can be a useful tool for 3D culture systems and various tissue engineering applications.

## Figures and Tables

**Table 1:** Primer sequences used for RT-PCR.

Symbol	Forward	Reverse
SOX2	GGCGGCAACCAGAAGAACAG	TCGATGAACGGCCGCTTCTC
KLF4	AGC CCCAAGATGCACAACTC	AGGACGAGGAAGAGGCAGAC
Wnt3a	TTCCTCAAGGACAAGTACGACA	GAAGTTGGGGGAGTTCTCATAG
Rex1	AGCCCAGCAGGCAGAAATGGAA	TGGTCAGTCTCACAGGGCACAT
c-MYC	TCGGACTCTCTGCTCTCCTC	CTTGTCGTTCTCCTCGGTGT
GAPDH	CAAGTTCCACGGCACGGTCA	CTCGGCACCAGCATCACCC

## Supplementary Materials

The following are available online at https://www.mdpi.com/1996-1944/12/15/2416/s1, Figure S1. Optical microscopy images of adipose-derived mesenchymal stem cells treated with astaxanthin at various concentrations of (a) 0 ng/ml, (b) 0.5 ng/ml, (c) 5 ng/ml, (d) 50 ng/ml, and € 500 ng/ml.
