# Supplementary material for: The Influence of Astaxanthin on the Proliferation of Adipose-derived Mesenchymal Stem Cells in Gelatin-Methacryloyl (GelMA) Hydrogels
Source: Materials (Basel). 2019 Jul 29;12(15):2416. doi: 10.3390/ma12152416 (PMC6696170; doi:10.3390/ma12152416)
Supplement: Supplementary file 1 [file materials-12-02416-s001.pdf]

materials

Supporting Information

The influence of Astaxanthin on Proliferation of Adipose-derived Mesenchymal Stem Cells in Gelatin-Methacryloyl (GelMA) Hydrogels

Bo Young Choi [1,2], Elna Paul Chalisserry [1,2], Myoung Hwan Kim [1,2], Hyun Wook Kang [1,2,3], Il-Whan Choi [4] and Seung Yun Nam [1,2,3,*]

1. Interdisciplinary Program of Biomedical Mechanical & Electrical Engineering, Pukyong National University, Busan, South Korea
2. Center for Marine-Integrated Biomedical Technology (BK21 Plus), Pukyong National University, Busan, South Korea
3. Department of Biomedical Engineering, Pukyong National University, Busan, South Korea;
4. Department of Microbiology, Inje University College of Medicine, Busan, South Korea

* Correspondence: synam@pknu.ac.kr; Tel.: +82-51-629-5776

Received: 29 June 2019; Accepted: 26 July 2019; Published: 28 July 2019

1. Cell morphology of ADMSCs in two-dimensional environments

The morphological characteristics of the adipose-derived mesenchymal stem cells with various concentrations of astaxanthin on day 3 are shown in Figure S1. In lieu with the above graph, the images confirmed that an increase in cell number was observed with healthy spindle shaped cells in (b) compared to (a). As shown in (d) and (e) of figures with high concentrations (50 and 500 ng/ml), lower cell numbers were indicated than (a) and (b).

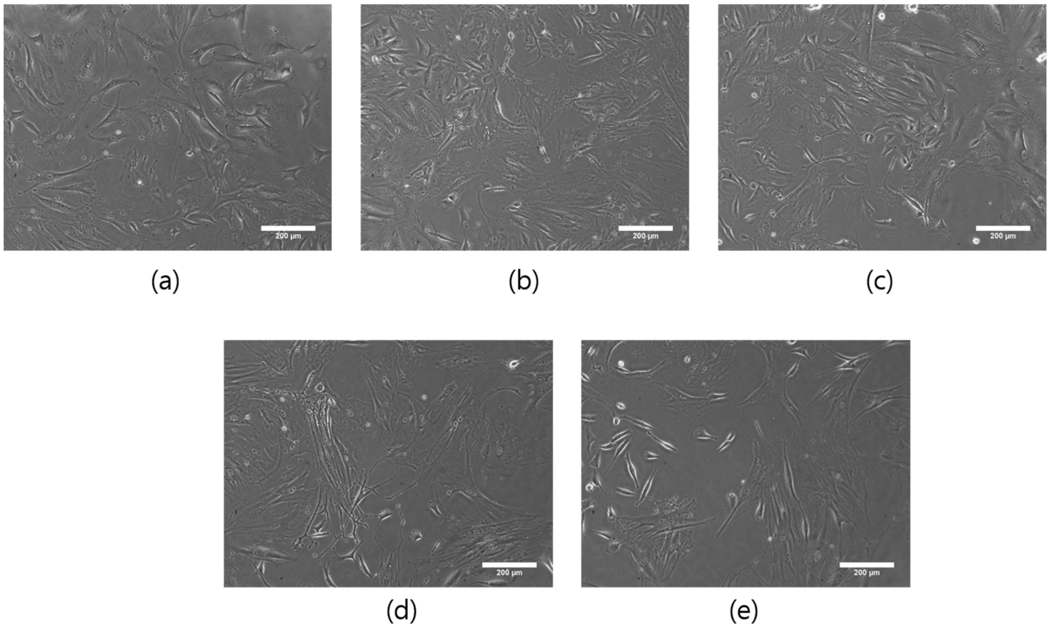

Figure S1. Optical microscopy images of adipose-derived mesenchymal stem cells treated with astaxanthin at various concentrations of (a) 0 ng/ml, (b) 0.5 ng/ml, (c) 5 ng/ml, (d) 50 ng/ml, and (e) 500 ng/ml. (scale = 200 μm)

© 2019 by the authors. Submitted for possible open access publication under the terms and conditions of the Creative Commons Attribution (CC BY) license (http://creativecommons.org/licenses/by/4.0/).
